# Screening and ARTP mutagenesis-based breeding of high-cellulase-producing strains

**DOI:** 10.1007/s42770-026-02086-5

**Published:** 2026-09-14

**Authors:** Laiyou Wang, Jintao Fang, Wei Chen, Yongpeng Jia, Liqing Guo, Shuang Cheng, Shuxian Guo, Bingbing Liu

**Affiliations:** 1 School of Biological and Chemical Engineering, Nanyang Institute of Technology, Nanyang, 473004 China; 2 Research Institute of Biological and Medical Sciences, Nanyang Institute of Technology, Nanyang, 473004 China; 3 Zhongjing College of Traditional Chinese Medicine and Health, Nanyang Vocational College, Nanyang, 473004 China; 4 Engineering Technology Research Center for Development and Utilization of Characteristic Medicinal Plants in Funiu Mountain, Nanyang, 474550 China; 5 Tiankang Biological Products Company Limited, Urumqi, 830001 China

**Keywords:** Soil microorganisms, Cellulose degrading, Mutagenesis, Atmospheric and Room-Temperature Plasma (ARTP), *Bacillus* sp.

## Abstract

While lignocellulose is an abundant renewable resource, the discovery of microorganisms capable of degrading lignocellulose is important for its full utilization. In this study, 26 soil samples were collected from Jigongshan National Nature Reserve, Xinyang, China. The Congo red staining method was used to isolate strains capable of degrading lignocellulose from the soil samples. A strain W-6-1, isolated from the soil samples was able to effectively degrade lignocellulose, with the filter paper activity (FPA) in the fermentation broth reaching 3.34 ± 0.32 U/mL. Integrating morphological traits, 16S rRNA phylogeny and physiological-biochemical assays, the isolate was identified as ***Bacillus *sp. W-6-1**. Three rounds of atmospheric and room-temperature plasma (ARTP) mutagenesis were employed to further enhance the lignocellulose-degrading ability of this strain. The best mutant (strain M3-35), which was selected from the surviving colonies, achieved an FPA of 6.11 ± 0.27 U/mL, representing an 83% increase over the wild-type strain W-6-1. Moreover, after multiple successive transfers, the FPA of strain M3-35 remained stable. The mutant M3-35 generated in this work provides a promising laboratory-derived candidate for exploring lignocellulose bioconversion.

**Supplementary Information:**

The online version contains supplementary material available at https://doi.org/10.1007/s42770-026-02086-5.

## Introduction

Plant cell walls are primarily composed of lignocellulose, which represents one of Earth’s most abundant and constantly renewable biomaterials [1, 2]. Lignocellulose forms the bulk of agricultural residues (such as straw and stover), forestry waste, dedicated energy crops, and even municipal green waste [3, 4]. Therefore, the efficient harnessing of lignocellulose via conversion to biofuels (e.g., cellulosic ethanol), biochemicals, or bioenergy is fundamentally crucial for the development of sustainable energy systems [5, 6]. Consequently, the efficient utilization of lignocellulose is a major research focus [7, 8].

Current lignocellulose degradation methods fall into three categories: physical, chemical, and biological. While physical and chemical methods exhibit certain advantages, they are also often time-consuming, labor-intensive, environmentally unfriendly, and costly [9, 10]. In contrast, biological degradation stands out as the most efficient, economical, and eco-friendly approach [11]. Therefore, strategies capable of harnessing microbial strains that possess both potent cellulose-degrading capabilities and high cellulase-producing potentials are garnering increasing scientific attention for maximizing the exploitation of lignocellulose [12–14].

Many microorganisms capable of degrading lignocellulose have already been reported [15, 16]. For example, Young et al. reported the production of an extracellular matrix by *Cellulomonas flavigena* KU (ATCC 53703), which enabled the degradation of microcrystalline cellulose [15]. Meanwhile, Zhang et al. enhanced the ability of *Trichoderma reesei* to synthesize cellulases by overexpressing a transcription factor [17]. However, most wild-type isolates hardly meet the requirements for practical industrial-scale applications because of their insufficient lignocellulose-degrading performance. Therefore, mutations must be physically or chemically induced in these wild-type strains to enhance their lignocellulose-degrading capacities. Atmospheric and room-temperature plasma (ARTP) mutagenesis is a strategy that employs helium radio frequency glow discharge plasma jets to induce microbial mutations [18]. Due to its consistency, high efficiency, nontoxicity, low cost, and environmental friendliness, ARTP mutagenesis compares favorably to other common mutation methods [19]. Bacterial, fungal, and microalgal strains have been successfully improved via ARTP mutagenesis, demonstrating its practical potential [20–22]. Furthermore, the production yields of various microbially generated products (e.g., 1,3-propanediol, hydrogen, high-acid protease, and lycopene) have been enhanced via this mutagenic technology [23–26]. Nevertheless, cases focusing on improving lignocellulose-degrading capacity of *Bacillus* strains using ARTP mutagenesis remain relatively limited.

In this study, soil samples were collected from Jigongshan National Nature Reserve, Xinyang, China. Microorganisms capable of cellulose degradation were isolated from the soil samples, and the microorganism with the strongest cellulose-degrading ability was selected. Subsequently, strain identification and ARTP mutagenesis were employed, and the mutant exhibiting the highest cellulose-degrading activity was selected.

## Materials and methods

### Treatment of soil samples

26 soil samples were acquired from Jigongshan National Nature Reserve, Xinyang, China (Table S1). A 10 mL enrichment medium (sodium carboxymethyl cellulose (CMC-Na) 5.0 g/L, (NH_4_)_2_SO_4_ 2.0 g/L, NaCl 0.5 g/L, K_2_HPO_4_ 1 g/L, MgSO_4_·7H_2_O 0.5 g/L, pH 7.0) was used to inoculate every 1 g soil sample (72 h, 200 rpm, 30 °C).

### Primary screening and purification

1 mL of the culture broth containing a soil sample was serially diluted with sterile water and spread onto a screening medium (CMC-Na 10.0 g/L, (NH_4_)_2_SO_4_ 2.0 g/L, NaCl 0.5 g/L, K_2_HPO_4_ 1 g/L, MgSO_4_·7H_2_O 0.5 g/L, Congo red 0.4 g/L, agar 20 g/L, pH 7.0). The agar plates were incubated at 30 °C for 72 h, and the plates were periodically examined to select colonies producing clear, transparent halos. Colonies without clear zones or with only very small clear zones were discarded. For colonies showing obvious clear zones on the same plate, only one representative isolate was retained when colonies possessed identical macroscopic morphology and consistent cell microscopic morphology, to eliminate duplicate strains. The chosen strains were then purified by streaking onto YPD medium (glucose 20.0 g/L, peptone 20.0 g/L, yeast extract 10.0 g/L, agar 20 g/L, pH 7.0) until single colonies were obtained. The single colonies were spotted onto the screening medium and incubated at 30 °C for 72 h. D/d ratios were then calculated using the clear zone diameter around each colony (D) and the colony diameter (d).

### Preparation of glucose standard curve

0, 0.2, 0.4, 0.6, 0.8, 1.0, 1.2, and 1.4 mL volumes of a 1 mg/mL glucose standard solution were pipetted into colorimetric tubes. The volumes in the tubes were each adjusted to 2 mL with distilled water, followed by the addition of 2 mL of DNS reagent. Each tube was then thoroughly mixed, boiled in a water bath for 5 min, and allowed to cool. Next, the volume in each tube was made up to 20 mL, and the absorbance (optical density, OD) of each mixture was evaluated at 540 nm using a spectrophotometer. A glucose standard curve was prepared by plotting the glucose mass (mg) on the x-axis and the OD on the y-axis, then fitting a regression equation (Fig. S1).

### Filter Paper Activity (FPA) assay

A single colony was inoculated into the fermentation medium for enzyme production (peptone 3.0 g/L, yeast extract 0.5 g/L, (NH_4_)_2_SO_4_ 2.0 g/L, KH_2_PO_4_ 4.0 g/L, CaCl_2_ 0.3 g/L, MgSO_4_·7H_2_O 0.3 g/L, CMC-Na 20 g/L) at 37 °C under shaking at 200 rpm for 24 h. Next, 5 mL of the fermentation broth extracted from the sample was added to each centrifuge tube. Centrifugation was performed at 5,000 rpm for 10 min, and the supernatant was collected as the crude enzyme solution for the assay. This assay was performed by using 0.5 mL of the crude enzyme solution to measure FPA values with 50 mg pieces of Xinhua filter paper as the substrate. The filter paper substrates were placed in 20 mL stoppered test tubes. 1.5 mL of citrate buffer and 0.5 mL of crude enzyme solution were then added to each test tube. Next, 1.5 mL DNS reagent was added to one tube as a control. The four tubes were preheated in a 50 °C water bath. The reaction then proceeded at 50 °C for 60 min. Afterward, the tubes were removed from the water bath, and 2.0 mL DNS reagent was immediately added to each sample. Subsequently, the tubes were then placed in a 100 °C water bath for 5 min, then cooled [27]. The volume of each tube was made up to 15 mL, and the OD of each sample at 540 nm was determined with a spectrophotometer. These OD values were then converted to glucose concentrations using the prepared glucose standard curve. The quantity of an enzyme that generates 1 µmol glucose per hour is defined as one enzyme activity unit (U). FPA values were calculated using the following formula:

$$\mathrm{FPA}\:=\:\mathrm G\left(\mathrm{mg}\right)\times\mathrm V\left(\mathrm{mL}\right)\times5.56/\left[\mathrm A\left(\mathrm{mL}\right)\times\mathrm B\left(\mathrm{mg}\right)\times\mathrm t\left(\mathrm h\right)\right]$$

Where G is the glucose amount; V is the final volume; t is the reaction time; A is the amount of enzyme added; B is the substrate mass; 5.56 is the number of µmol in 1 mg glucose.

### DNA extraction, PCR amplification, and phylogenetic analyses

A genomic DNA extraction kit (TIANGEN, Beijing) was employed to extract genomic DNA from the strain. The genomic DNA of the isolate underwent PCR amplification using universal primers for bacterial 16S rDNA. A universal DNA purification kit (TIANGEN, Beijing) was employed to purify and recover the PCR products, which were then sequenced. The obtained 16S rRNA gene sequence was submitted to GenBank with accession number PZ794417, and the sequence is now publicly available. Sequence-based identification was conducted using the EzBioCloud database (https://www.ezbiocloud.net/), and MEGA 7.0 software was used to construct a phylogenetic tree to determine the genetic relationships among the strains.

### Phenotypic analyses

Growth characteristics were determined in Luria-Bertani (LB) medium. The temperature-growth profile was tested across 5–50 °C at 5 °C intervals. The pH tolerance was evaluated from pH 3.0 to 11.0 at 0.5 pH-unit increments. NaCl tolerance was assayed at NaCl concentrations ranging from 0% to 7% (w/v) in 1% increments. Bacterial growth was monitored by measuring optical density at 600 nm (OD₆₀₀). Biochemical features were analysed using API 20E and API 50CH strips (bioMérieux) according to Logan and Berkeley [28].

### ARTP mutagenesis and screening

A 10 µL seed culture suspension was evenly spread on a sterile metal plate for treatment with pure helium plasma under a radio frequency power input of 120 W and 10 SLM helium flow rate using an ARTP Breeding Mutagenesis Machine (ARTP-M model, Yuanqing Tianmu Biotechnology Co., Ltd., Wuxi, China) [29]. After mutagenesis, the metal plate was immediately placed in a sterilized tube containing 1 mL of LB medium. Each LB medium plate was spread with an appropriate dilution of the obtained cell suspension, followed by 24 h of cultivation at 37 °C. After manual colony counting, ARTP treatment lethality was calculated using the following equation:

$$\mathrm{Lethality}\;(\%)\;=\;(\mathrm{Control}\;\mathrm{colonies}--\mathrm{Survival}\;\mathrm{colonies})/\mathrm{Control}\;\mathrm{colonies}\times100$$

### Genetic stability of mutants

The mutant strains were first cultured on plates with LB solid medium at 37 °C for 24 h. Then, new LB solid medium plates were streaked with several colonies and subcultured for 24 h. Subculturing was performed for each selected mutant strain 10 times (in other words, for 10 generations). Each mutant subculture then underwent inoculation in fermentation medium and 24 h of incubation at 37 °C. The resulting culture broth was utilized as the crude enzyme solution for FPA quantification. FPA monitoring was performed across the ten generations to evaluate mutant genetic stability.

### Statistical analysis

Triplicate experimental measurements were performed in all cases. Basic data were processed using Microsoft Excel 2016, figures were produced with GraphPad Prism 6.0, and significance analysis was conducted with SPSS 19.0. One-way ANOVA followed by Tukey’s post hoc test was used to assess significant differences among groups.

## Results

### Initial screening

**Fig. 1:**
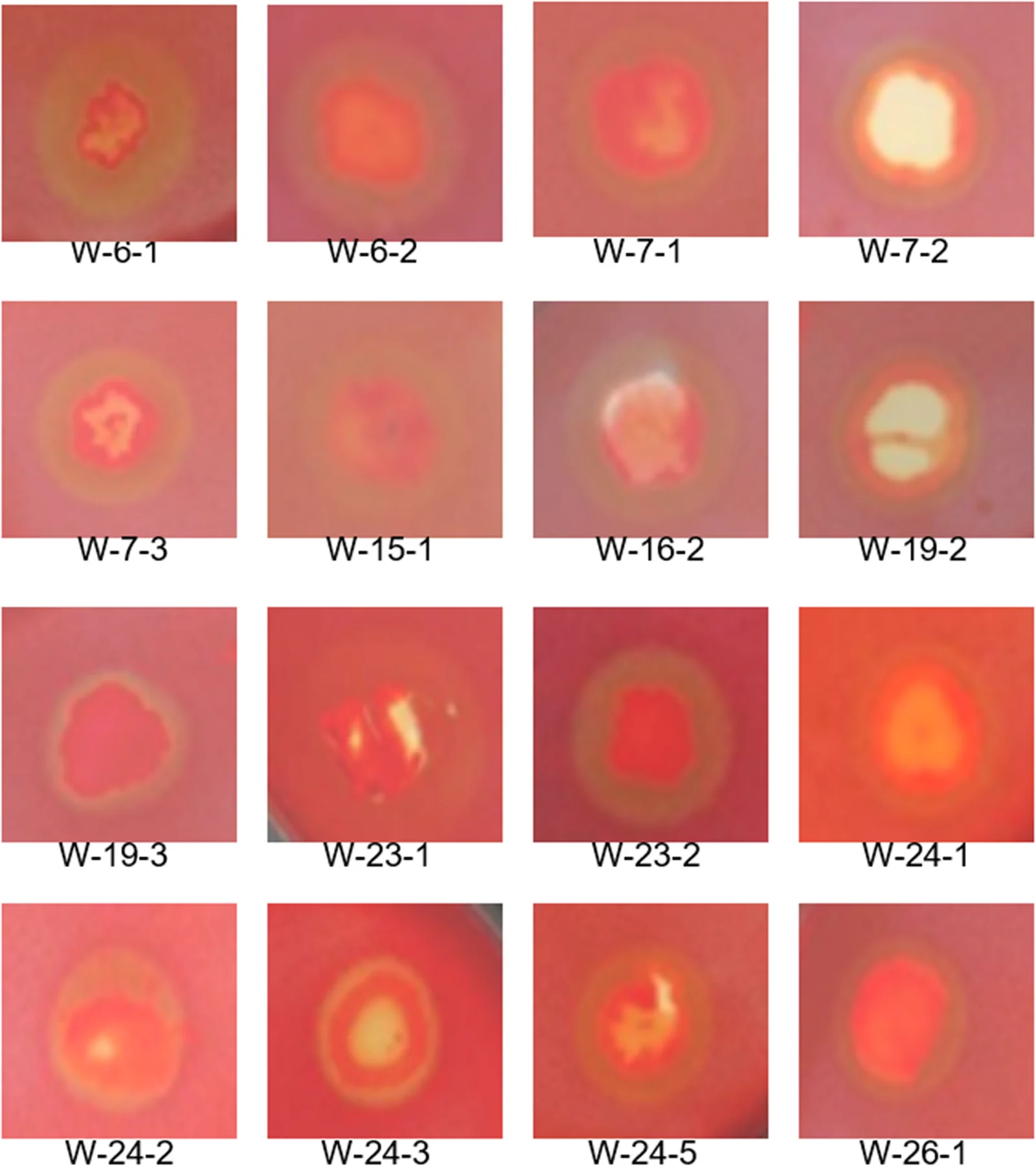
Congo red staining test results of the sixteen colonies obtained from initial screening. The text below each staining result indicates the strain number

After enrichment cultivation, the samples were preliminarily screened using the Congo red plate staining method. The total number of initial colonies on agar plates was not recorded in this work. Duplicate isolates with identical colony-cell morphological features were removed, and 16 unique isolates were finally retained. These 16 strains were isolated, purified, and then re-inoculated onto plates containing screening medium (Fig. 1). Their colony diameters and clear zone diameters were subsequently measured (Table S2). As presented in Table S2, five strains (W-6-1, W-6-2, W-7-3, W-23-1 and W-23-2) exhibited a significantly higher ratio of clear zone diameter to colony diameter compared with other strains (*p* < 0.05). These five strains were chosen for secondary screening. Subsequently, these five strains were grown in the fermentation medium for 24 h, and after each culture broth was centrifuged, their FPA values were measured. As shown in Table 1, strain W-6-1 exhibited the highest FPA. Therefore, W-6-1 was chosen as the starting strain for the subsequent ARTP mutagenesis.

**Table 1:** FPA of five strains obtained from initial screening

Strains	FPA(U/mL)
W-6-1	3.34 ± 0.32^a^
W-6-2	3.06 ± 0.12^ab^
W-7-3	2.34 ± 0.23^c^
W-23-1	2.45 ± 0.36^c^
W-23-2	3.11 ± 0.37^ab^

Note: Data are shown as mean ± standard deviation from three biological replicates. Values marked with different superscript lowercase letters are significantly different at *p* < 0.05, as determined by one-way ANOVA followed by Tukey’s post-hoc test

### Identification of W-6-1

**Fig. 2:**
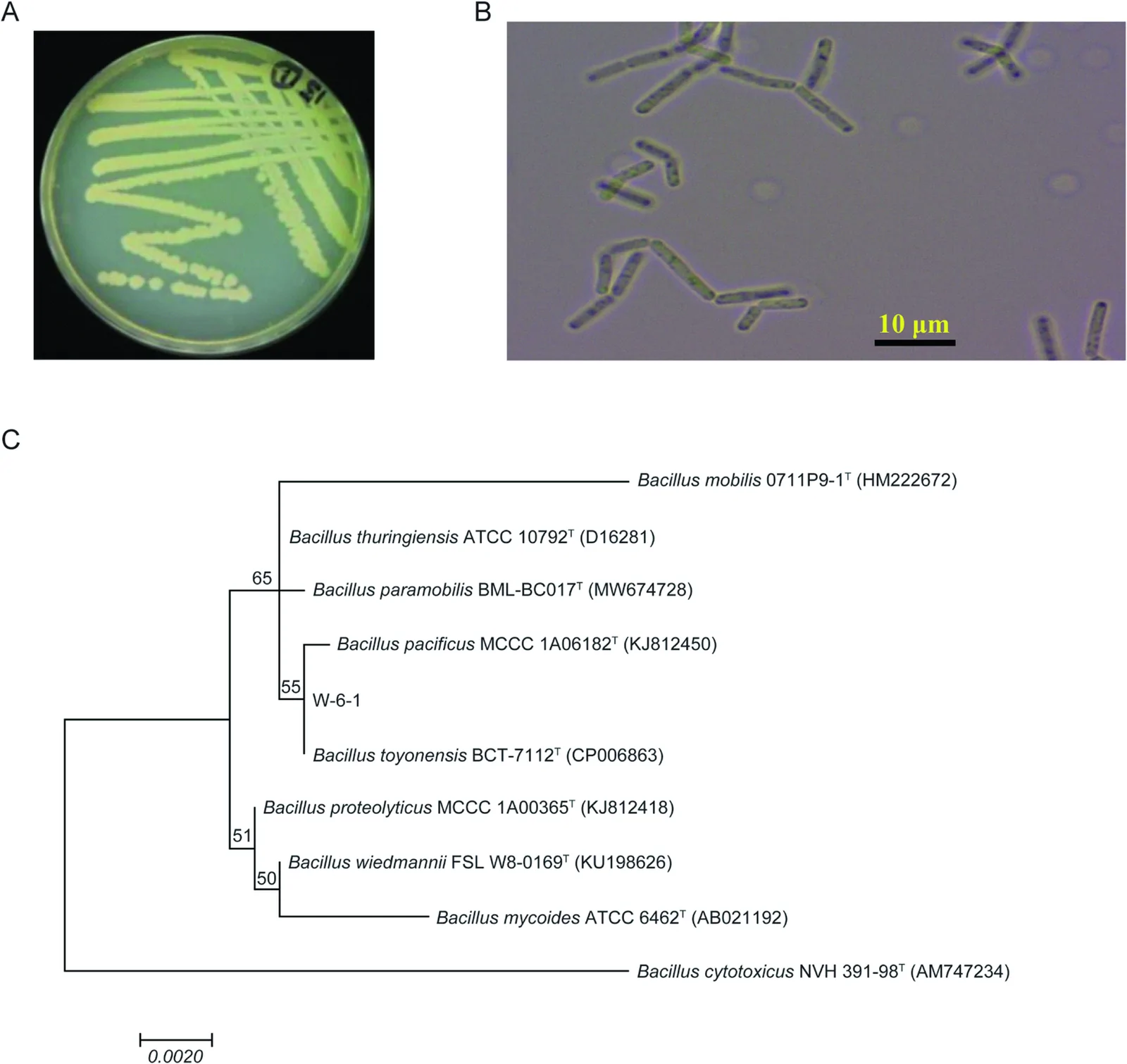
Morphological and phylogenetic evaluations of strain W-6-1. Morphologies of (**A**) colonies and (**B**) cells of the strain W-6-1. (**C**) Neighbor-joining phylogenetic tree based on 16S rDNA sequences showing the relationship of strain W-6-1 to the closely related strains

Morphological observation, 16S rRNA-gene-based phylogenetic analysis, and comprehensive phenotypic characterization were combined to determine the taxonomic status of strain W-6-1. An LB plate was used to culture W-6-1 cells (24 h, 37 °C ) prior to morphological observation. The resulting W-6-1 colony had a creamy, white appearance with irregular edges and featureless surface bulges (Fig. 2A). When viewed under a light microscope, the W-6-1 cells were straight, rod-shaped bacilli with widths of about 0.7–1.0 μm and lengths of roughly 2–5 μm; these cells were present as either single rods or short chains (Fig. 2B). The 16S rRNA gene sequence of strain W-6-1 was obtained for phylogenetic analysis. Sequence similarity analysis revealed that strain W-6-1 shared 100.00% sequence identity with *Bacillus toyonensis* BCT-7112^T^, and 99.93% sequence similarity with *Bacillus pacificus* MCCC 1A06182^T^. Phylogenetic trees constructed based on the 16S rRNA sequences indicated that strain W-6-1 clustered into the same branch as *B. toyonensis* (Fig. 2C).

Phenotypic characterization demonstrated that strain W-6-1 exhibited identical growth characteristics, including temperature tolerance (10–45 °C, optimum 35 °C), NaCl tolerance (0–5%, w/v), and pH adaptability (pH 5.0–9.5, optimum pH 6.5), to *B. toyonensis* BCT-7112ᵀ (Table 2) [30]. Nevertheless, obvious differential biochemical phenotypes were observed in API 20E and API 50CH tests (Table 2). Strain W-6-1 was positive for lysine decarboxylase and ornithine decarboxylase, whereas these two reactions were negative for the type strain. In the API 50CH system, several carbohydrate-utilization patterns also differentiated strain W-6-1 from *B. toyonensis* BCT-7112ᵀ (Table 2). Compared with the type strain, strain W-6-1 was negative for D-ribose, methyl-α-D-glucopyranoside, amygdalin, D-saccharose (sucrose) and D-turanose. By contrast, D-mannose, arbutin and starch showed weak-positive (+w) reactions in strain W-6-1. All remaining API 20E and API 50CH phenotypic traits were consistent between the two strains.

**Table 2:** Comparison of phenotypic properties between strain W-6-1 and the type strain *B. toyonensis* BCT-7112ᵀ

Characteristic	*B. toyonensis* BCT-7112^T^	*Bacillus* sp. W-6-1
Growth temperature range (°C)	10–45	10–45
Optimal growth temperature (°C)	35	35
Salinity tolerance range (% NaCl)	0–5	0–5
Optimal growth salinity (% NaCl)	0	0
pH tolerance range	5–9.5	5–9.5
Optimal pH	6.5	6.5
API 20E		
lysine decarboxylase	**−**	+
ornithine decarboxylase	**−**	+
API 50CH		
D-Ribose	+	**−**
D-Mannose	**−**	+w
Methyl-α-D-glucopyranoside	+	**−**
Amygdalin	+w	**−**
Arbutin	+	+w
D-Saccharose (sucrose)	+	**−**
Starch	+	+w
D-Turanose	+	**−**

Note: +, positive; −, negative; +w, weak-positive. The remaining test items of API 20E and API 50CH exhibited identical results between strain W-6-1 and *B. toyonensis* BCT-7112ᵀ. Data for *B. toyonensis* BCT-7112ᵀ were obtained from the reference

Although 16 SrRNA gene sequence analysis showed 100.00% sequence identity with *B. toyonensis* BCT-7112ᵀ, several diagnostic biochemical differences were detected between the isolate and the reference type strain. Considering these phenotypic variations, definitive species assignment could not be achieved merely based on the available evidence. Accordingly, the isolate was designated *Bacillus* sp. W-6-1.

### Lethality rate of *Bacillus* sp. W-6-1 with ARTP mutagenesis

**Fig. 3:**
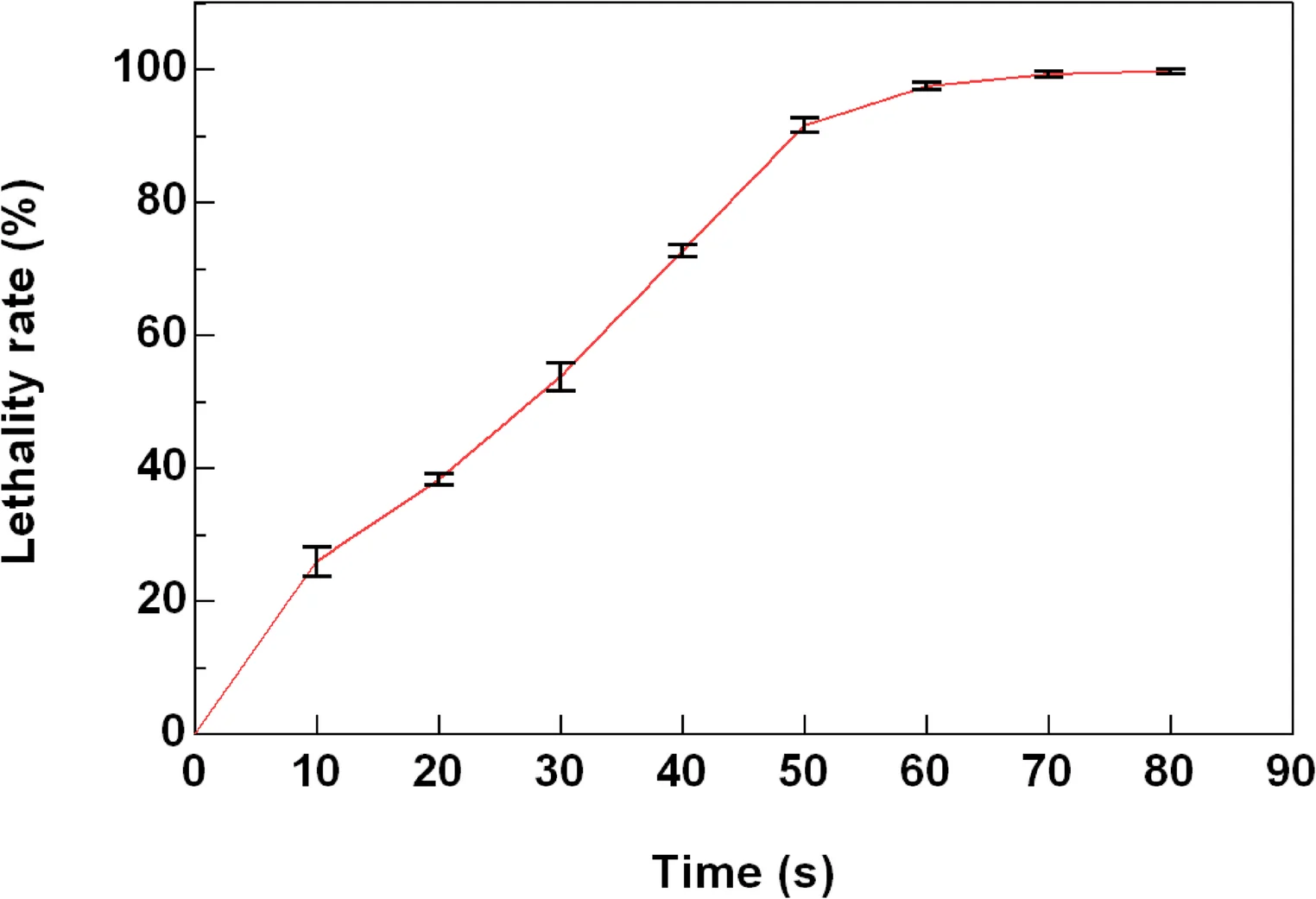
Effects of the exposure time of ARTP treatment on the lethality of the strain W-6-1

During ARTP mutagenesis treatment, the ARTP dose determines the strain lethality rate [31]. In turn, the strain lethality rate is closely related to the positive-mutation rate [32]. To efficiently generate mutations, the lethality rate should be about 95%. Therefore, the lethality rates of strain W-6-1 under various ARTP exposure times were evaluated to identify the optimal treatment time. As shown in Fig. 3, as the exposure time was increased, the lethality rate also rose. Almost full lethality was achieved after 80 s, which was too long. However, a lethality rate of 97.49 ± 0.33% was attained after 60 s of exposure. Therefore, a 60 s ARTP exposure time was chosen for ARTP mutagenesis in this work.

### Mutagenesis and screening

**Fig. 4:**
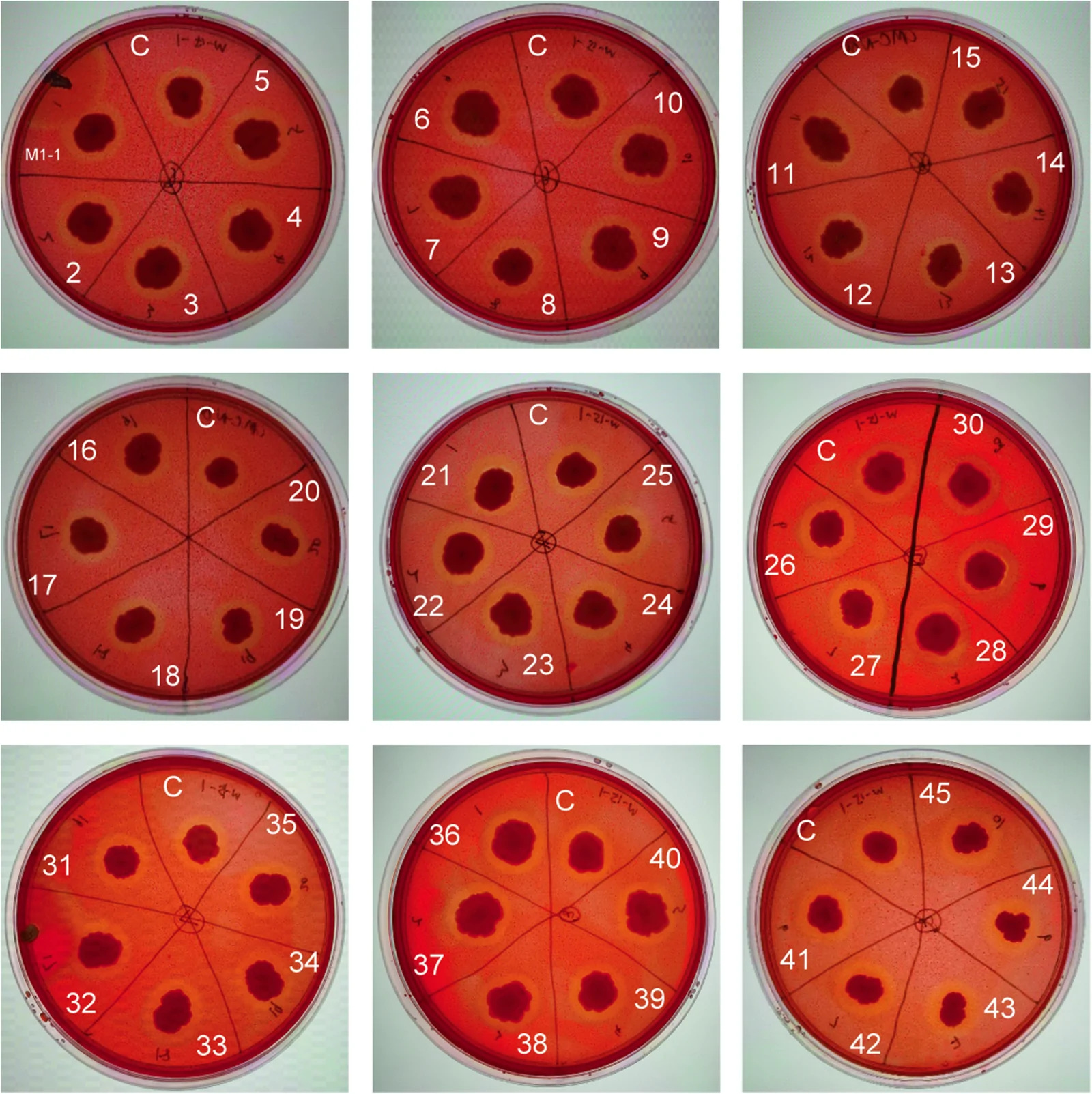
Congo red staining test results of the strains obtained from first-round mutagenesis. C represents the strain W-6-1. Numbers 1 to 45 correspond to strains M1-1 through M1-45, respectively

The first-round mutagenesis results of strain W-6-1 are shown in Fig. 4 and Table S3. 26 positive mutants and 19 negative mutants were achieved, resulting in a positive mutation rate of 57.78%. To minimize experimental error, strain W-6-1 was spotted on each plate. Each strain’s D/d ratio was normalized to the D/d ratio of W-6-1 on the same plate to obtain its relative ratio. Mutants whose relative ratios were assigned superscript “a” exhibited statistically significantly higher halo-to-colony ratios on CMC-Congo red screening plates. Nevertheless, the halo-to-colony ratio is only a semi-quantitative plate-based proxy indicator and cannot perfectly predict actual cellulase activity under liquid fermentation conditions. Among these mutants with the highest statistically ranked relative ratios (superscript “a”), three isolates with stable colony morphology and robust growth status (M1-41, M1-43, and M1-44) were selected for further quantitative filter-paper activity (FPA) measurement, rather than testing all mutants possessing this statistical ranking. As shown in Table 3, strain M1-44 exhibited the highest FPA. The FPA of M1-44 was 1.24 times that of strain W-6-1.

**Table 3:** FPA of the strains screened after each round of mutagenesis

	Strains	FPA(U/mL)
1st round	W-6-1^p^	3.34 ± 0.32^d^
	M1-41	3.86 ± 0.67^cd^
	M1-43	3.92 ± 0.17^c^
	M1-44	4.15 ± 0.28^c^
2nd round	M1-44^p^	4.15 ± 0.28^c^
	M2-2	5.23 ± 0.78^b^
	M2-3	4.62 ± 0.18^bc^
	M2-24	4.83 ± 0.49^bc^
3rd round	M2-2^p^	5.23 ± 0.78^b^
	M3-8	5.89 ± 0.13^ab^
	M3-35	6.11 ± 0.27^a^
	M3-63	5.67 ± 0.36^ab^

“^p^” denotes the parental strain used in each round of mutagenesis. “^a−d^” denote the significant difference (*P* < 0.05). There is no significant difference between samples marked with the same letter, and there is significant difference between samples marked with different letters

**Fig. 5:**
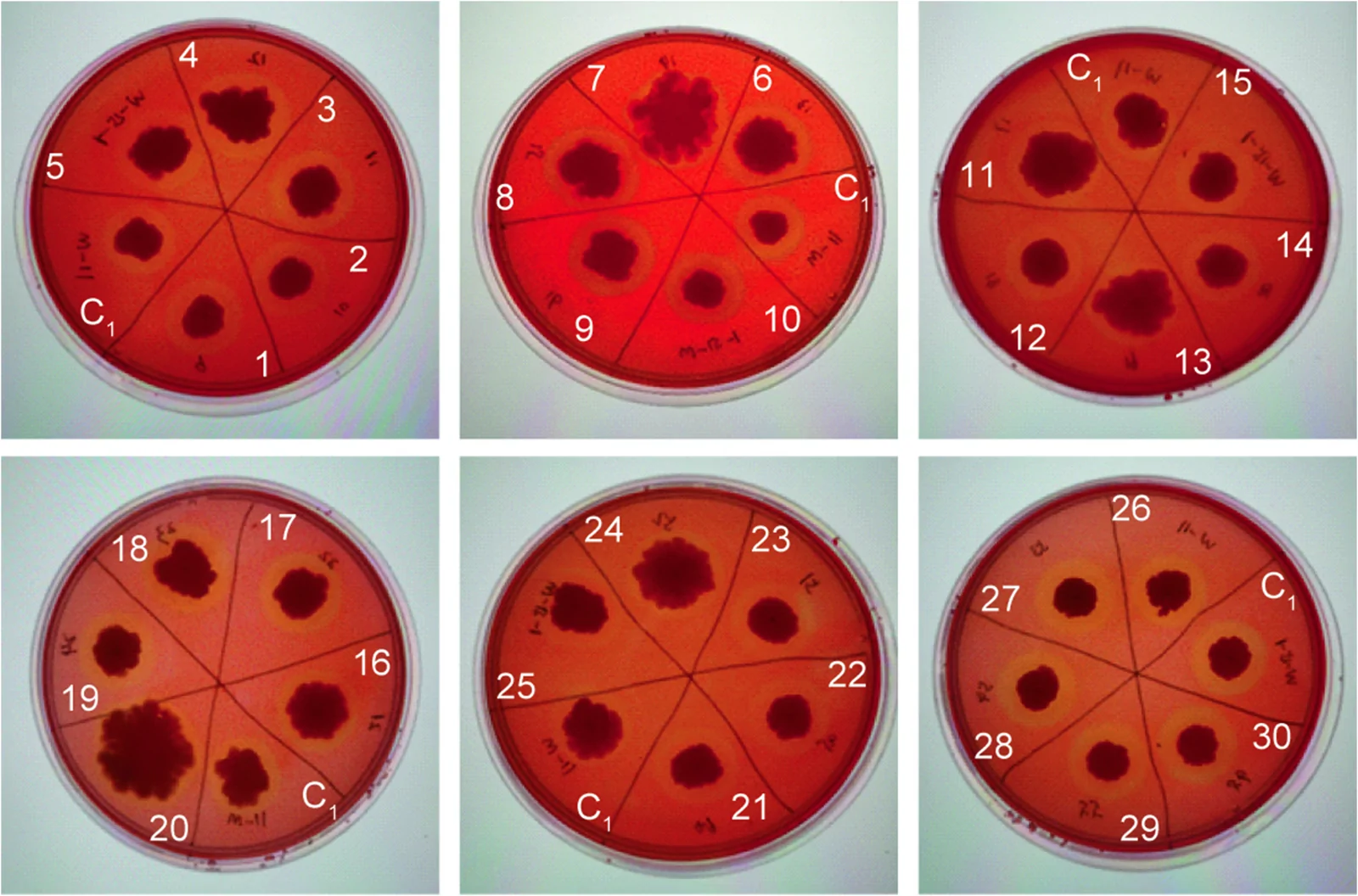
Congo red staining test results of the strains obtained from second-round mutagenesis. C_1_ represents the strain M1-44. Numbers 1 to 30 correspond to strains M2-1 through M2-30, respectively

Subsequently, mutant M1-44 was subjected to a second round of mutagenesis, as shown in Fig. 5 and Table S4. There were 7 positive mutants and 23 negative mutants, indicating a positive mutation rate of 23.33%. Strain M1-44 was spotted on each plate. Each strain’s D/d ratio was normalized to the D/d ratio of M1-44 on the same plate to obtain its relative ratio. Among the resulting mutants, only M2-2 and M2-24 possessed relative ratios bearing superscript “a”, representing the statistically highest values (Table S4). Although mutant M2-3 did not obtain this statistical marker, it showed a comparatively high D/d ratio together with favorable colony growth and was therefore also selected for FPA quantification. FPA assays were performed on these three candidate isolates (M2-2, M2-3 and M2-24). Their FPA values were measured. As shown in Table 3, strain M2-2 exhibited the highest FPA. The FPA of M2-2 was 1.26 times that of strain M1-44.

**Fig. 6:**
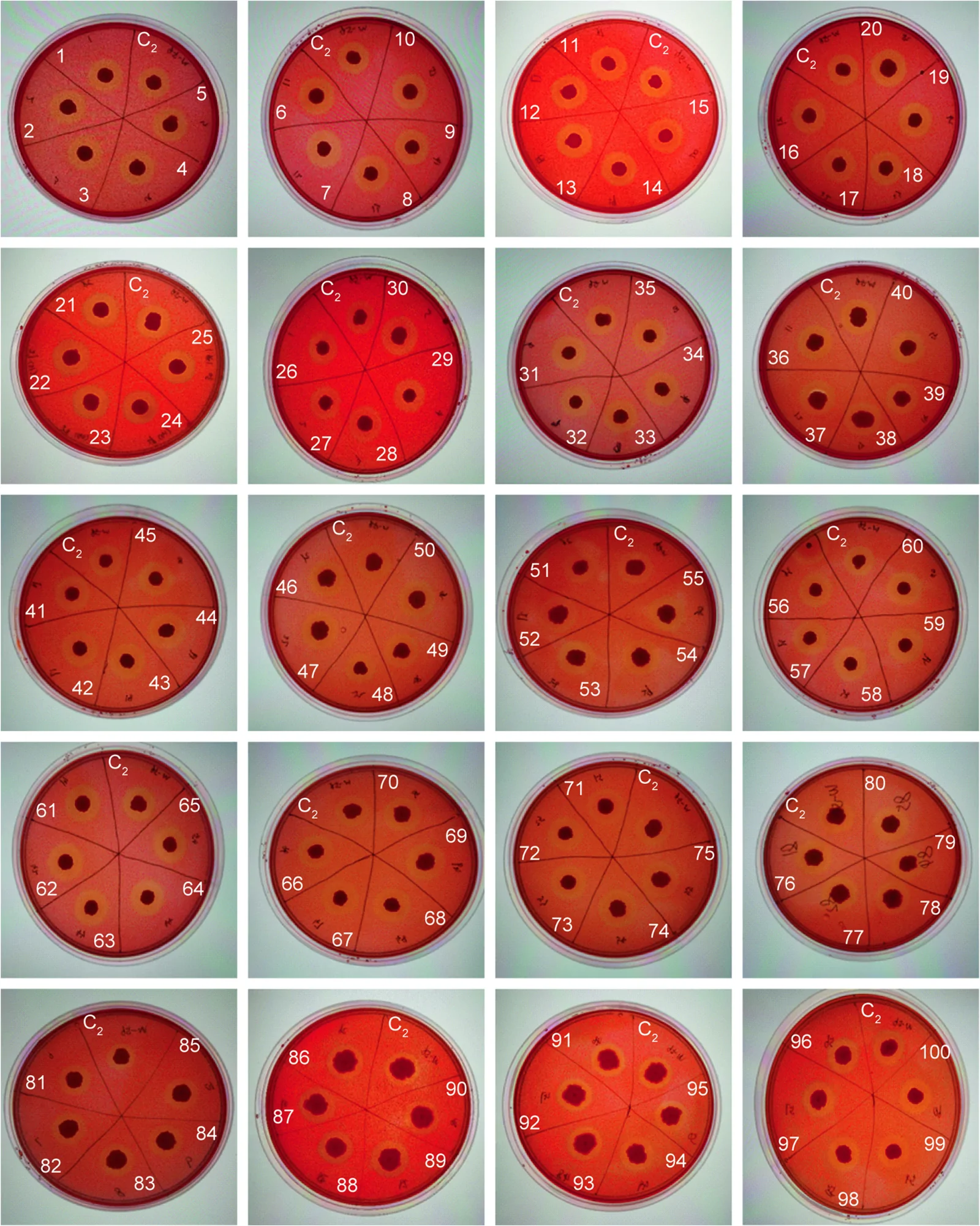
Congo red staining test results of the strains obtained from third-round mutagenesis. C_2_ represents the strain M2-2. Numbers 1 to 100 correspond to strains M3-1 through M3-100, respectively

Mutant M2-2 was subjected to a third round of mutagenesis, as shown in Fig. 6 and Table S5. There were 69 positive mutants and 31 negative mutants, resulting in a positive mutation rate of 69.00%. Strain M2-2 was spotted on each plate. Each strain’s D/d ratio was normalized to the D/d ratio of M2-2 on the same plate to obtain its relative ratio. Among mutants that possessed relative ratios bearing superscript “a”, M3-8, M3-35 and M3-63 were chosen for FPA measurement. As shown in Table 3, strain M3-35 exhibited the highest FPA. The FPA of M3-35 was 1.17 times that of strain M2-2. In addition, the FPA of M3-35 was 1.83 times that of strain W-6-1.

### Mutant genetic stability

**Fig. 7:**
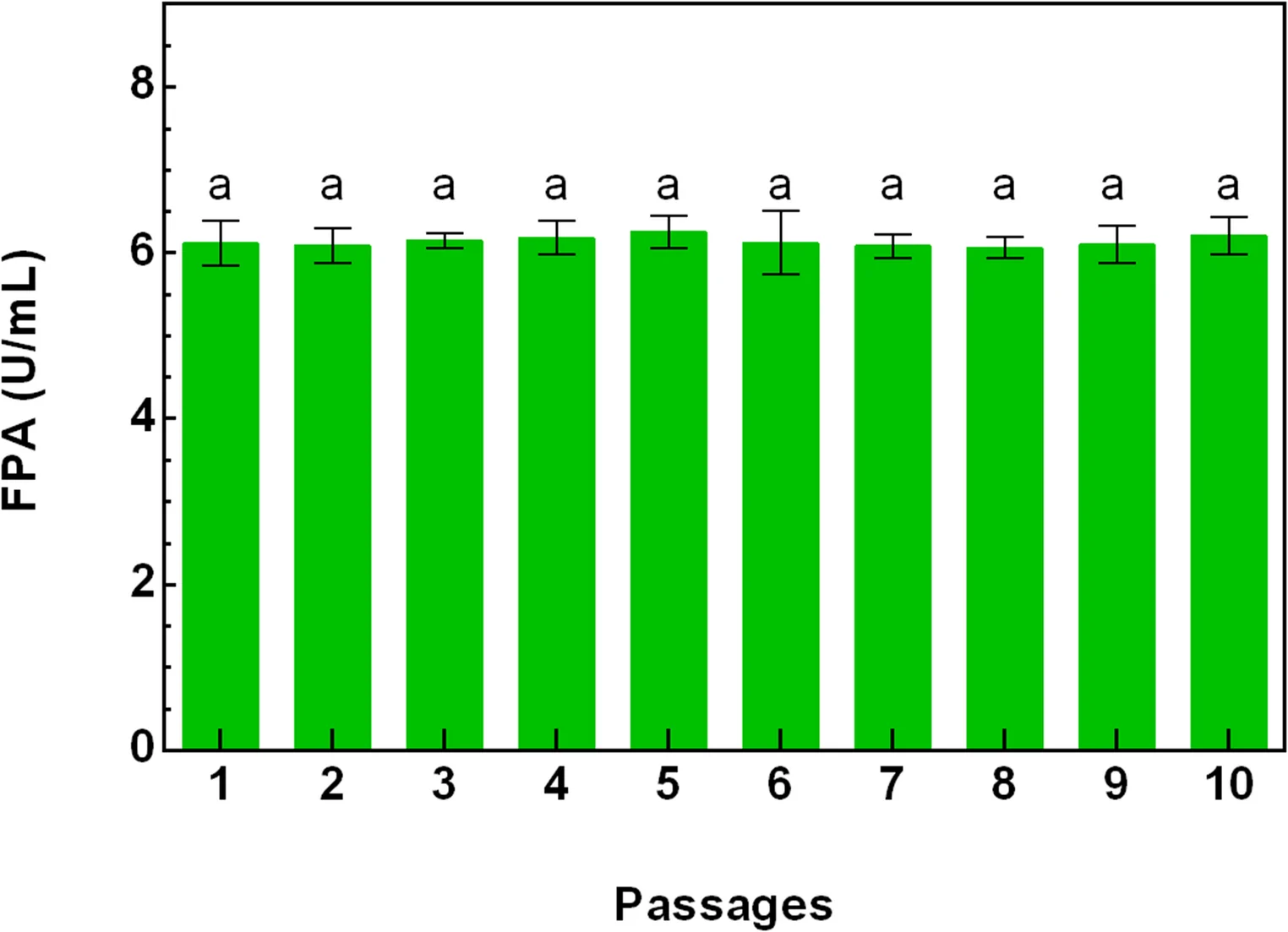
The FPA values in the first and tenth generations of strain M3-35. “a” indicates the results of statistical analysis. Statistical analysis showed that there was no significant difference (*p* > 0.05) among the ten serial passages

The industrial application of genetically mutated strains relies on achieving mutations with good genetic stability. Therefore, the genetic stability of strain M3-35 was evaluated after undergoing mutagenesis for ten generations (Fig. 7). Statistical analysis was further performed on the data from the ten serial passages. No significant differences (*p* > 0.05) were observed among different generations. The FPA values of the first and tenth generations were comparable, which strongly confirmed the excellent genetic stability of the mutant strain (Fig. 7).

## Discussion

The production of fermentable sugar via the transformation of lignocellulosic biomass using cellulose enzymes shows good promise [33]. Notably, microorganisms in the soil can generate hydrolytic enzymes crucially involved in degrading cellulose. Many soil microorganisms with the potential for achieving good cellulose degradation performance have been isolated in recent years. For example, *B. subtilis K1*, *Raoultella* sp. S12, and *Bosea* sp. FBZP-16 have all been reported to show excellent performance as cellulase-producing bacteria [34–36]. In this study, 16 cellulase-producing strains were isolated from 26 soil samples. Fourteen of the isolates were obtained from soil beneath trees and shrubs, and two isolates were obtained from soil beneath weeds. This is potentially because leaf litter and dead twigs from trees and shrubs are a relatively rich source of nutrients for cellulolytic bacteria. Of these 16 isolates, strain W-6-1 exhibited the highest cellulase-producing capacity. Although phylogenetic analysis confirmed that strain W-6-1 shared 100.00% 16S rRNA gene sequence identity with *B. toyonensis* BCT-7112ᵀ, distinct phenotypic differences precluded precise species-level identification, and the isolate was therefore classified as *Bacillus* sp. Nevertheless, previous studies have demonstrated that strains belonging to *B. toyonensis* possess prominent cellulose-degrading ability. For instance, Vyasa et al. isolated *B. toyonensis* LC3B1 from the gut of white grubs and verified its strong cellulolytic activity [37]. Collectively, these observations suggest that *Bacillus* strains phylogenetically affiliated with *Bacillus toyonensis* constitute valuable candidates for investigating lignocellulose bioconversion. To further improve the cellulase yield of the isolated strain, three rounds of mutagenesis were performed on *Bacillus* sp. W-6-1 to screen high-yield mutant strains.

ARTP is a novel cell mutagenesis technique that can effectively induce DNA damage, leading to an enhanced mutation frequency. Moreover, this mutagenesis method does not produce toxic or hazardous substances, providing a significant advantage compared to traditional ultraviolet irradiation or chemical mutagens [38]. Different microbial species have different optimal ARTP exposure times. For example, the optimal mutagenesis time for enhancing the lactic acid production of a spore-forming lactic acid bacterium was reported to be 90 s, at which point the lethality was 90%–95%. Brewer’s yeast (*Saccharomyces cerevisiae*) was treated with an exposure time of 120 s to yield desirable mutants, resulting in a lethality of 96.3% [39, 40]. Consistent with related studies, this study found that the lethality rate of *Bacillus* sp. W-6-1 increased with increasing treatment time, reaching 97.49 ± 0.33% at 60 s of mutagenesis. The positive mutation rate is also a decisive factor affecting the efficacy of strain mutagenesis. Studies have shown that higher positive mutation rates are attained under lethality rates in the range of 90%–95% [41]. The positive mutation rate was observed to substantially vary in this work, even for the same mutagenesis time. For example, the positive mutation rates from three rounds of mutagenesis in this study were 57.78%, 23.33%, and 69.00%. Finally, mutant M3-35 with enhanced filter-paper activity was obtained after three cycles of ARTP treatment.

To further evaluate the effectiveness of our iterative mutagenesis strategy, we compared the present work with previously reported ARTP-mediated strain-improvement cases (Table 4). As summarized in Table 4, most reported ARTP-based strain-improvement studies adopt only one or two rounds of mutagenesis, and the magnitude of trait enhancement varies greatly depending on microbial host, target product and screening criteria. Moderate performance improvements are frequently obtained from single-round ARTP mutagenesis, for example, a 17.6% increase in pullulan yield in *Aureobasidium pullulans*, 54.1% enhancement of DHA biosynthesis in *Schizochytrium* sp., and an 82% elevation in phospholipase D activity in *Streptomyces hiroshimensis* [42–44]. A small subset of studies achieved multi-fold improvements via single-round mutagenesis, and iterative two-round ARTP treatments have also been applied for further yield boosting [19, 45]. Distinct from most published studies relying on one- or two-cycle ARTP mutagenesis, the present study performed three successive rounds of ARTP treatment on *Bacillus* sp. W-6-1 and achieved an 83% increase in filter paper activity. Our work provides a reference case for applying multi-cycle iterative ARTP mutagenesis to improve lignocellulose-degrading enzyme activity in *Bacillus*.

**Table 4:** Comparison of performance improvements obtained by mutagenesis among different microbial strains

Strain	Target metabolite/activity	Mutagenesis rounds	Increase relative to parent strain	Reference
*Schizochytrium* sp. PKU#Mn4	Docosahexaenoic acid production	2	54.1%	[44]
*Pseudomonas* sp. L01	Rhamnolipids production	1	2.7-fold	[19]
*Streptomyces roseosporus* L2796	Daptomycin production	2	58.33%	[45]
*Aureobasidium pullulans* RM1603	Pullulan production	1	17.6%	[42]
*Bacillus coagulans* WT-03	Probiotic performance	1	15.2%	[46]
*Streptomyces* *hiroshimensis* SK43.001	Phospholipase D activity	1	82%	[43]
*Bacillus thuringiensis* X023	Cry protein production	2	61%.	[22]
*Actinoplanes deccanensis* OE-R1	Fidaxomicin production	1	5.5-fold	[47]
*Brevibacillus sp.* SPR20	Anti-MRSA activity	1	improved	[48]
*Saccharopolyspora spinosa* CCTCC M206084	Spinosad production	1	1.8-fold	[31]
*Enterococcus faecalis*	Digestive enzyme activity	1	180% (α-amylase), 30% (lipase), > 40% (neutral protease)	[49]
*Aspergillus niger* CGMCC 3.3783	Dextranase DEX production	1	30.6-fold	[50]
*Stenotrophomonas maltophilia* SE5	Selenium nanoparticles (SeNPs) production	1	40%	[51]
*Paenarthrobacter nitroguajacolicus* LDT1	CMCase activity	1	21.72%	[52]
*Bacillus* sp. W-6-1	Filter paper activity	3	83%	This study

Accumulating multi-omics studies have uncovered the molecular mechanisms underlying ARTP-driven microbial mutation and phenotypic remodelling [51, 53]. ARTP treatment generates abundant single-nucleotide polymorphisms (SNPs) and insertion-deletion (InDel) mutations; these genetic changes frequently occur within gene regulatory regions and signal-transduction pathways rather than creating entirely novel functional proteins, leading to genome-wide transcriptional reprogramming of target strains [52]. For example, comparative genomic and transcriptomic analyses of an ARTP-mutated *Paenarthrobacter nitroguajacolicus* revealed that mutations enriched in regulatory regions triggered coordinated up-regulation of multiple lignocellulose-degradation-related genes and improved degradation capacity [52]. Whole-genome resequencing and transcriptome profiling of ARTP-derived *Sphingomonas paucimobilis* mutant indicated that mutations in two-component system pathways reshaped global gene expression, up-regulated synthase-coding genes and suppressed lyase-encoding genes, jointly contributing to higher gellan-gum production [53]. Likewise, ARTP mutagenesis in *Stenotrophomonas maltophilia* up-regulated key genes related to substrate uptake, redox homeostasis and biosynthesis pathways and improved both synthetic performance and stress tolerance [51]. By analogy to these findings, the elevated filter paper activity of mutant M3-35 is most likely caused by ARTP-induced mutations in regulatory elements or signal-transduction systems, which remodel the transcription of cellulase-related genes. Nevertheless, the exact causal mutation sites remain unknown in our study, as whole-genome resequencing, transcriptomic or comparative proteomic analyses have not yet been performed. Comparative proteomics between the wild-type strain and mutant M3-35 would offer further mechanistic insights into the phenotypic alteration, and such multi-omics investigation will be considered in our future research.

Although mutant M3-35 displayed improved filter paper activity and maintained stable phenotypic performance over ten successive generations under laboratory culture conditions, several limitations of this study should be acknowledged. First, ten-generation serial subculture cannot fully guarantee the long-term genetic stability required for continuous industrial fermentation. Second, this study only assessed enzyme activity under laboratory conditions. Critical evaluations, including actual lignocellulosic biomass degradation capacity, enzyme tolerance under industrial-simulated conditions, systematic fermentation optimization and pilot-scale fermentation validation, are still absent. Accordingly, over-optimistic claims for direct industrial applicability are inappropriate at this stage. Mutant M3-35 should be regarded as a promising laboratory-derived candidate strain for further research rather than a ready-to-use industrial strain.

In future research, we intend to perform whole-genome sequencing and functional annotation for wild-type *Bacillus* sp. W-6-1 and mutant M3-35 to mine cellulase-encoding genes. Comparative genomics will be used to screen candidate genetic mutations, while comparative proteomics will identify differentially expressed proteins contributing to enhanced cellulase activity. Directed-evolution strategies will be adopted to further improve the catalytic performance of key cellulase candidates. In addition, further experiments will focus on evaluating its degradation performance on real lignocellulosic feedstock, optimizing fermentation parameters and conducting scale-up trials to explore its practical application potential. The mutant strain and relevant functional gene resources obtained in this work are valuable and deserve further investigation.

## Conclusion

In this study, a cellulase-producing bacterial strain W-6-1 was successfully isolated from soil samples collected from Jigongshan National Nature Reserve, Xinyang, China. Based on morphological observation and 16S rRNA gene phylogenetic analysis, strain W-6-1 exhibited 100.00% sequence identity with the type strain *B. toyonensis* BCT-7112ᵀ. However, distinct phenotypic discrepancies in API 20E and API 50CH biochemical characteristics were detected between strain W-6-1 and the reference strain, which precluded accurate species-level classification. Accordingly, the isolate was finally identified as *Bacillus* sp. W-6-1.

Using the original strain *Bacillus* sp. W-6-1 as the parent strain, three successive rounds of ARTP iterative mutagenesis were conducted for strain improvement. After systematic screening, a high-yield mutant strain M3-35 with significantly enhanced cellulase activity was obtained. The mutant M3-35 exhibited prominently improved filter paper cellulase activity and excellent genetic stability after continuous subculture, demonstrating that multi-cycle ARTP mutagenesis is an effective strategy for enhancing the cellulolytic capacity of *Bacillus* strains.

Overall, this study provides a promising microbial resource for the bioconversion of lignocellulosic biomass. Further multi-omics analysis will be performed to elucidate the molecular regulatory mechanism responsible for the improved cellulase activity of the mutant strain.

## Supplementary Information

Below is the link to the electronic supplementary material.

Supplementary Material 1 Table S1 Detailed information of soil samples. Table S2 Colony diameters and clear zone diameters of 16 strains obtained from initial screening. Table S3 Colony diameters and clear zone diameters of strains obtained from first-round mutagenesis. Table S4 Colony diameters and clear zone diameters of strains obtained from second-round mutagenesis. Table S5 Colony diameters and clear zone diameters of strains obtained from third-round mutagenesis. Fig. S1 Glucose standard curve for DNS test.

## Data Availability

The 16S rRNA gene sequence of strain W-6-1 was deposited in GenBank with accession number PZ794417, and the sequence is publicly accessible. Other data generated in this study are available from the corresponding author upon reasonable request.
