# Supplementary material for: Screening and ARTP mutagenesis-based breeding of high-cellulase-producing strains
Source: Braz J Microbiol. 2026 Sep 14;57(1):272. doi: 10.1007/s42770-026-02086-5 (PMC13575069; doi:10.1007/s42770-026-02086-5)
Supplement: Supplementary file 1 [file 42770_2026_2086_MOESM1_ESM.docx]

*Brazilian Journal of Microbiology*

**Screening and ARTP mutagenesis-based breeding of high-cellulase-producing strains**

Laiyou Wang^1,2^, Jintao Fang^3,4^, Wei Chen^1^, Yongpeng Jia^1^, Liqing Guo^5^, Shuang Cheng^1,2^, Shuxian Guo^1^ & Bingbing Liu^1,2^ *

1, School of Biological and Chemical Engineering, Nanyang Institute of Technology, Nanyang 473004, China

2, Research Institute of Biological and Medical Sciences, Nanyang Institute of Technology, Nanyang 473004, China

3, Zhongjing College of Traditional Chinese Medicine and Health，Nanyang Vocational College, Nanyang 473004, China

4, Engineering Technology Research Center for Development and Utilization of Characteristic Medicinal Plants in Funiu Mountain, Nanyang 474550, China

5, Tiankang Biological Products Company Limited, Urumqi 830001, China

***Corresponding author:**

Prof. Bingbing Liu

Research Institute of Biological and Medical Sciences, Nanyang Institute of Technology, Nanyang 473004, China

Email: liubingbing200666@163.com

**Table S1** Detailed information of soil samples

| Sample ID | Vegetation cover | Altitude (m) | Latitude (°N) and longitude (°E) |
| --- | --- | --- | --- |
| 1 | Shrub | 174.2 | 31.8027; 114.0542 |
| 2 | Weed | 215.9 | 31.8280; 114.0509 |
| 3 | Tree | 211.6 | 31.8281; 114.0511 |
| 4 | Shrub | 421.8 | 31.8401; 114.0553 |
| 5 | Weed | 445.5 | 31.8045; 114.0546 |
| 6 | Shrub | 243.13 | 31.8555; 114.0299 |
| 7 | Tree | 118.5 | 31.8404; 113.9962 |
| 8 | Tree | 128 | 31.8410; 113.9961 |
| 9 | Shrub | 263.6 | 31.8306; 114.0691 |
| 10 | Shrub | 285.7 | 31.8299; 114.0692 |
| 11 | Tree | 326.7 | 31.8244; 114.0656 |
| 12 | Weed | 332.2 | 31.8239; 114.0656 |
| 13 | Weed | 401.2 | 31.8187; 114.0726 |
| 14 | Tree | 390.9 | 31.8187; 114.0726 |
| 15 | Tree | 405.5 | 31.8189; 114.073 |
| 16 | Tree | 481.5 | 31.8165; 114.0750 |
| 17 | Tree | 476.1 | 31.8167; 114.0748 |
| 18 | Tree | 636.8 | 31.8103; 114.0804 |
| 19 | Weed | 645.1 | 31.8083; 114.0794 |
| 20 | Weed | 660.2 | 31.8075; 114.0792 |
| 21 | Weed | 702.9 | 31.8037; 114.0737 |
| 22 | Tree | 696.9 | 31.8016; 114.0739 |
| 23 | Tree | 750.9 | 31.8013; 114.0728 |
| 24 | Shrub | 716.96 | 31.8006; 114.0744 |
| 25 | Shrub | 712.8 | 31.8034; 114.0748 |
| 26 | Shrub | 550.3 | 31.8176; 114.0785 |

**Fig. S1** Glucose standard curve for DNS test.

**Table S2** Colony diameters and clear zone diameters of 16 strains obtained from initial screening.

| Strains | Colony diameters (d, cm) | Clear zone diameters (D, cm) | Ratio D/d |
| --- | --- | --- | --- |
| W-6-1 | 0.78 | 1.78 | 2.28^a^ |
| W-6-2 | 1.11 | 1.98 | 1.78^a^ |
| W-7-1 | 1.24 | 1.77 | 1.43^b^ |
| W-7-2 | 1.34 | 1.65 | 1.23^c^ |
| W-7-3 | 0.95 | 1.59 | 1.67^a^ |
| W-15-1 | 1.20 | 1.77 | 1.48^b^ |
| W-16-2 | 1.14 | 1.72 | 1.51^b^ |
| W-19-2 | 1.32 | 1.72 | 1.30^b^ |
| W-19-3 | 1.13 | 1.40 | 1.24^c^ |
| W-23-1 | 1.26 | 2.01 | 1.60^a^ |
| W-23-2 | 0.92 | 1.69 | 1.84^a^ |
| W-24-1 | 1.23 | 1.60 | 1.30^b^ |
| W-24-2 | 1.10 | 1.45 | 1.32^b^ |
| W-24-3 | 1.15 | 1.35 | 1.17^c^ |
| W-24-5 | 1.09 | 1.26 | 1.16^c^ |
| W-26-1 | 1.18 | 1.55 | 1.31^b^ |

“^a−c^” denote the significant difference (P < 0.05). There is no significant difference between samples marked with the same letter, and there is significant difference between samples marked with different letters.

**Table S3** Colony diameters and clear zone diameters of strains obtained from first-round mutagenesis.

| **Strains** | **Colony diameters (d, cm)** | **Clear zone diameters (D, cm)** | **Ratio D/d** | **Relative ratio** |
| --- | --- | --- | --- | --- |
| M1-1 | 1.22 | 1.66 | 1.36 | 0.96^d^ |
| M1-2 | 1.26 | 1.76 | 1.39 | 0.99^c^ |
| M1-3 | 1.29 | 1.71 | 1.33 | 0.94^d^ |
| M1-4 | 1.24 | 1.69 | 1.37 | 0.97^d^ |
| M1-5 | 1.35 | 1.83 | 1.35 | 0.96^d^ |
| W-6-1 | 1.19 | 1.67 | 1.41 | 1^c^ |
| M1-6 | 1.40 | 1.84 | 1.32 | 1.07^b^ |
| M1-7 | 1.33 | 1.70 | 1.28 | 1.04^c^ |
| M1-8 | 1.14 | 1.55 | 1.36 | 1.1^b^ |
| M1-9 | 1.31 | 1.74 | 1.32 | 1.07^b^ |
| M1-10 | 1.29 | 1.72 | 1.34 | 1.09^b^ |
| W-6-1 | 1.34 | 1.64 | 1.23 | 1^c^ |
| M1-11 | 1.52 | 1.79 | 1.18 | 0.94^d^ |
| M1-12 | 1.25 | 1.52 | 1.22 | 0.97^d^ |
| M1-13 | 1.22 | 1.47 | 1.21 | 0.97^d^ |
| M1-14 | 1.27 | 1.46 | 1.15 | 0.92^d^ |
| M1-15 | 1.20 | 1.41 | 1.17 | 0.94^d^ |
| W-6-1 | 1.11 | 1.38 | 1.25 | 1^c^ |
| M1-16 | 1.23 | 1.66 | 1.35 | 0.9^d^ |
| M1-17 | 1.18 | 1.59 | 1.35 | 0.9^d^ |
| M1-18 | 1.17 | 1.56 | 1.34 | 0.9^d^ |
| M1-19 | 0.98 | 1.52 | 1.56 | 1.05^c^ |
| M1-20 | 1.14 | 1.50 | 1.32 | 0.89^d^ |
| W-6-1 | 0.86 | 1.29 | 1.49 | 1^c^ |
| M1-21 | 1.20 | 1.72 | 1.44 | 1.08^b^ |
| M1-22 | 1.19 | 1.67 | 1.40 | 1.05^c^ |
| M1-23 | 1.24 | 1.72 | 1.38 | 1.04^c^ |
| M1-24 | 1.15 | 1.64 | 1.42 | 1.07^b^ |
| M1-25 | 1.07 | 1.66 | 1.56 | 1.17^b^ |
| W-6-1 | 1.09 | 1.46 | 1.33 | 1^c^ |
| M1-26 | 1.14 | 1.66 | 1.46 | 1.01^c^ |
| M1-27 | 1.12 | 1.77 | 1.58 | 1.09^b^ |
| M1-28 | 1.23 | 1.77 | 1.44 | 0.99^c^ |
| M1-29 | 1.24 | 1.76 | 1.42 | 0.98^d^ |
| M1-30 | 1.25 | 1.76 | 1.41 | 0.97^d^ |
| W-6-1 | 1.18 | 1.71 | 1.45 | 1^c^ |
| M1-31 | 0.99 | 1.52 | 1.54 | 1.1^b^ |
| M1-32 | 1.12 | 1.66 | 1.49 | 1.05^c^ |
| M1-33 | 1.23 | 1.75 | 1.42 | 1.01^c^ |
| M1-34 | 1.13 | 1.65 | 1.46 | 1.04^c^ |
| M1-35 | 1.12 | 1.54 | 1.37 | 0.97^d^ |
| W-6-1 | 1.14 | 1.60 | 1.41 | 1^c^ |
| M1-36 | 1.38 | 1.85 | 1.34 | 0.99^c^ |
| M1-37 | 1.37 | 2.01 | 1.47 | 1.08^b^ |
| M1-38 | 1.26 | 1.91 | 1.51 | 1.11^b^ |
| M1-39 | 1.15 | 1.69 | 1.48 | 1.09^b^ |
| M1-40 | 1.33 | 1.84 | 1.38 | 1.01^c^ |
| W-6-1 | 1.16 | 1.58 | 1.36 | 1^c^ |
| M1-41 | 0.96 | 1.58 | 1.64 | 1.66^a^ |
| M1-42 | 0.99 | 1.61 | 1.63 | 1.65^a^ |
| M1-43 | 1.02 | 1.68 | 1.65 | 1.66^a^ |
| M1-44 | 0.98 | 1.70 | 1.74 | 1.75^a^ |
| M1-45 | 1.05 | 1.66 | 1.59 | 1.6^a^ |
| W-6-1 | 1.49 | 1.48 | 0.99 | 1^c^ |

“^a-d^” denote the significant difference (P < 0.05). There is no significant difference between samples marked with the same letter, and there is significant difference between samples marked with different letters.

**Table S4** Colony diameters and clear zone diameters of strains obtained from second-round mutagenesis.

| **Strains** | **Colony diameters (d, cm)** | **Clear zone diameters (D, cm)** | **Ratio D/d** | **Relative ratio** |
| --- | --- | --- | --- | --- |
| M2-1 | 1.07 | 1.76 | 1.65 | 1.09^b^ |
| M2-2 | 0.92 | 1.83 | 1.99 | 1.32^a^ |
| M2-3 | 1.19 | 2.05 | 1.73 | 1.15^b^ |
| M2-4 | 1.55 | 2.11 | 1.36 | 0.9^c^ |
| M2-5 | 1.34 | 1.97 | 1.47 | 0.98^c^ |
| M1-44 | 1.19 | 1.79 | 1.51 | 1^c^ |
| M2-6 | 1.31 | 1.99 | 1.52 | 0.79^d^ |
| M2-7 | 2.00 | 2.44 | 1.22 | 0.63^e^ |
| M2-8 | 1.43 | 2.15 | 1.50 | 0.78^d^ |
| M2-9 | 1.34 | 1.97 | 1.47 | 0.76^d^ |
| M2-10 | 1.07 | 1.62 | 1.51 | 0.78^d^ |
| M1-44 | 0.82 | 1.59 | 1.93 | 1^c^ |
| M2-11 | 1.74 | 2.26 | 1.30 | 0.76^d^ |
| M2-12 | 1.18 | 1.94 | 1.64 | 0.96^c^ |
| M2-13 | 1.76 | 2.03 | 1.15 | 0.68^e^ |
| M2-14 | 1.13 | 1.73 | 1.53 | 0.9^c^ |
| M2-15 | 1.15 | 1.53 | 1.33 | 0.78^d^ |
| M1-44 | 1.11 | 1.87 | 1.70 | 1^c^ |
| M2-16 | 1.51 | 2.10 | 1.39 | 0.8^d^ |
| M2-17 | 1.25 | 2.08 | 1.67 | 0.96^c^ |
| M2-18 | 1.41 | 2.04 | 1.45 | 0.83^d^ |
| M2-19 | 1.21 | 1.96 | 1.62 | 0.93^c^ |
| M2-20 | 2.42 | 2.68 | 1.11 | 0.64^e^ |
| M1-44 | 1.25 | 2.17 | 1.74 | 1^c^ |
| M2-21 | 1.07 | 1.85 | 1.73 | 1.1^b^ |
| M2-22 | 1.17 | 1.92 | 1.64 | 1.05^b^ |
| M2-23 | 1.82 | 2.20 | 1.20 | 0.77^d^ |
| M2-24 | 0.99 | 1.90 | 1.92 | 1.22^a^ |
| M2-25 | 1.32 | 1.99 | 1.50 | 0.96^c^ |
| M1-44 | 1.38 | 2.17 | 1.57 | 1^c^ |
| M2-26 | 1.01 | 1.89 | 1.87 | 0.97^c^ |
| M2-27 | 1.04 | 1.88 | 1.81 | 0.94^c^ |
| M2-28 | 2.42 | 1.70 | 0.70 | 0.36^e^ |
| M2-29 | 0.95 | 1.94 | 2.03 | 1.05^b^ |
| M2-30 | 1.03 | 1.87 | 1.82 | 0.94^c^ |
| M1-44 | 1.00 | 1.92 | 1.93 | 1^c^ |

“^a-e^” denote the significant difference (P < 0.05). There is no significant difference between samples marked with the same letter, and there is significant difference between samples marked with different letters.

**Table S5** Colony diameters and clear zone diameters of strains obtained from third-round mutagenesis.

| **Strains** | **Colony diameters (d, cm)** | **Clear zone diameters (D, cm)** | **Ratio D/d** | **Relative ratio** |
| --- | --- | --- | --- | --- |
| M3-1 | 0.70 | 1.54 | 2.20 | 1.14^b^ |
| M3-2 | 0.73 | 1.54 | 2.10 | 1.1^b^ |
| M3-3 | 0.74 | 1.61 | 2.17 | 1.13^b^ |
| M3-4 | 0.71 | 1.49 | 2.11 | 1.1^b^ |
| M3-5 | 0.73 | 1.48 | 2.04 | 1.06^b^ |
| M2-2 | 0.77 | 1.47 | 1.92 | 1^c^ |
| M3-6 | 0.76 | 1.61 | 2.11 | 1.04^c^ |
| M3-7 | 0.69 | 1.62 | 2.34 | 1.15^b^ |
| M3-8 | 0.65 | 1.68 | 2.61 | 1.28^a^ |
| M3-9 | 0.64 | 1.54 | 2.40 | 1.18^b^ |
| M3-10 | 0.71 | 1.59 | 2.25 | 1.11^b^ |
| M2-2 | 0.65 | 1.31 | 2.03 | 1^c^ |
| M3-11 | 0.71 | 1.51 | 2.13 | 0.94^c^ |
| M3-12 | 0.73 | 1.61 | 2.20 | 0.97^c^ |
| M3-13 | 0.74 | 1.65 | 2.23 | 0.98^c^ |
| M3-14 | 0.70 | 1.65 | 2.35 | 1.04^c^ |
| M3-15 | 0.65 | 1.57 | 2.40 | 1.06^b^ |
| M2-2 | 0.65 | 1.46 | 2.26 | 1^c^ |
| M3-16 | 0.70 | 1.65 | 2.34 | 1.04^c^ |
| M3-17 | 0.70 | 1.61 | 2.30 | 1.03^c^ |
| M3-18 | 0.74 | 1.59 | 2.15 | 0.96^c^ |
| M3-19 | 0.72 | 1.59 | 2.23 | 0.99^c^ |
| M3-20 | 0.78 | 1.73 | 2.22 | 0.99^c^ |
| M2-2 | 0.64 | 1.43 | 2.24 | 1^c^ |
| M3-21 | 0.78 | 1.64 | 2.11 | 1.04^c^ |
| M3-22 | 0.80 | 1.70 | 2.12 | 1.05^b^ |
| M3-23 | 0.91 | 1.64 | 1.81 | 0.9^c^ |
| M3-24 | 0.77 | 1.59 | 2.07 | 1.03^c^ |
| M3-25 | 0.75 | 1.70 | 2.26 | 1.12^b^ |
| M2-2 | 0.79 | 1.59 | 2.02 | 1^c^ |
| M3-26 | 0.64 | 1.47 | 2.29 | 1.11^b^ |
| M3-27 | 0.70 | 1.47 | 2.09 | 1.02^c^ |
| M3-28 | 0.79 | 1.63 | 2.07 | 1^c^ |
| M3-29 | 0.74 | 1.59 | 2.14 | 1.04^c^ |
| M3-30 | 0.83 | 1.82 | 2.20 | 1.07^b^ |
| M2-2 | 0.70 | 1.45 | 2.06 | 1^c^ |
| M3-31 | 0.73 | 1.62 | 2.23 | 1.12^b^ |
| M3-32 | 0.65 | 1.42 | 2.18 | 1.1^b^ |
| M3-33 | 0.76 | 1.60 | 2.12 | 1.06^b^ |
| M3-34 | 0.67 | 1.64 | 2.46 | 1.24^a^ |
| M3-35 | 0.66 | 1.75 | 2.65 | 1.33^a^ |
| M2-2 | 0.76 | 1.51 | 1.99 | 1^c^ |
| M3-36 | 0.87 | 1.72 | 1.98 | 1.02^c^ |
| M3-37 | 0.89 | 1.75 | 1.98 | 1.02^c^ |
| M3-38 | 1.07 | 1.78 | 1.66 | 0.86^d^ |
| M3-39 | 0.80 | 1.53 | 1.93 | 0.99^c^ |
| M3-40 | 0.75 | 1.60 | 2.14 | 1.1^b^ |
| M2-2 | 0.86 | 1.67 | 1.94 | 1^c^ |
| M3-41 | 0.66 | 1.60 | 2.43 | 0.97^c^ |
| M3-42 | 0.74 | 1.68 | 2.28 | 0.91^c^ |
| M3-43 | 0.68 | 1.69 | 2.48 | 0.99^c^ |
| M3-44 | 0.64 | 1.69 | 2.64 | 1.06^b^ |
| M3-45 | 0.68 | 1.63 | 2.40 | 0.96^c^ |
| M2-2 | 0.61 | 1.52 | 2.50 | 1^c^ |
| M3-46 | 0.87 | 1.61 | 1.85 | 1.03^c^ |
| M3-47 | 0.99 | 1.69 | 1.72 | 0.95^c^ |
| M3-48 | 0.66 | 1.41 | 2.15 | 1.19^b^ |
| M3-49 | 0.73 | 1.65 | 2.26 | 1.25^a^ |
| M3-50 | 0.84 | 1.74 | 2.07 | 1.15^b^ |
| M2-2 | 0.85 | 1.52 | 1.80 | 1^c^ |
| M3-51 | 0.67 | 1.45 | 2.17 | 1.24^a^ |
| M3-52 | 0.92 | 1.73 | 1.88 | 1.07^b^ |
| M3-53 | 0.88 | 1.61 | 1.83 | 1.04^c^ |
| M3-54 | 0.90 | 1.69 | 1.87 | 1.07^b^ |
| M3-55 | 0.84 | 1.68 | 2.01 | 1.15^b^ |
| M2-2 | 0.84 | 1.47 | 1.75 | 1^c^ |
| M3-56 | 0.66 | 1.57 | 2.39 | 1.11^b^ |
| M3-57 | 0.65 | 1.61 | 2.46 | 1.15^b^ |
| M3-58 | 0.65 | 1.61 | 2.48 | 1.15^b^ |
| M3-59 | 0.69 | 1.69 | 2.44 | 1.14^b^ |
| M3-60 | 0.64 | 1.67 | 2.62 | 1.22^a^ |
| M2-2 | 0.64 | 1.38 | 2.15 | 1^c^ |
| M3-61 | 0.75 | 1.68 | 2.23 | 1.1^b^ |
| M3-62 | 0.77 | 1.68 | 2.18 | 1.08^b^ |
| M3-63 | 0.66 | 1.71 | 2.60 | 1.28^a^ |
| M3-64 | 0.69 | 1.56 | 2.25 | 1.11^b^ |
| M3-65 | 0.72 | 1.48 | 2.06 | 1.02^c^ |
| M2-2 | 0.75 | 1.52 | 2.03 | 1^c^ |
| M3-66 | 0.72 | 1.55 | 2.16 | 1.09^b^ |
| M3-67 | 0.81 | 1.67 | 2.06 | 1.04^c^ |
| M3-68 | 0.67 | 1.63 | 2.44 | 1.23^a^ |
| M3-69 | 0.78 | 1.77 | 2.27 | 1.14^b^ |
| M3-70 | 0.82 | 1.59 | 1.93 | 0.98^c^ |
| M2-2 | 0.73 | 1.44 | 1.98 | 1^c^ |
| M3-71 | 0.76 | 1.74 | 2.29 | 1.19^b^ |
| M3-72 | 0.67 | 1.57 | 2.33 | 1.21^a^ |
| M3-73 | 0.70 | 1.62 | 2.33 | 1.2^a^ |
| M3-74 | 0.85 | 1.74 | 2.06 | 1.07^b^ |
| M3-75 | 0.80 | 1.74 | 2.18 | 1.13^b^ |
| M2-2 | 0.86 | 1.65 | 1.93 | 1^c^ |
| M3-76 | 0.89 | 1.70 | 1.90 | 0.98^c^ |
| M3-77 | 0.91 | 1.65 | 1.81 | 0.93^c^ |
| M3-78 | 1.03 | 1.62 | 1.57 | 0.81^d^ |
| M3-79 | 0.74 | 1.56 | 2.10 | 1.08^b^ |
| M3-80 | 0.87 | 1.74 | 2.02 | 1.04^c^ |
| M2-2 | 0.63 | 1.22 | 1.94 | 1^c^ |
| M3-81 | 0.78 | 1.40 | 1.80 | 1.1^b^ |
| M3-82 | 0.80 | 1.44 | 1.79 | 1.1^b^ |
| M3-83 | 0.94 | 1.43 | 1.52 | 0.93^c^ |
| M3-84 | 0.88 | 1.48 | 1.67 | 1.03^c^ |
| M3-85 | 0.85 | 1.44 | 1.70 | 1.05^b^ |
| M2-2 | 0.96 | 1.56 | 1.63 | 1^c^ |
| M3-86 | 1.07 | 1.75 | 1.64 | 0.88^d^ |
| M3-87 | 0.95 | 1.67 | 1.77 | 0.95^c^ |
| M3-88 | 0.90 | 1.68 | 1.87 | 1^c^ |
| M3-89 | 1.02 | 1.73 | 1.69 | 0.91^c^ |
| M3-90 | 1.09 | 1.66 | 1.52 | 0.82^d^ |
| M2-2 | 0.86 | 1.60 | 1.86 | 1^c^ |
| M3-91 | 1.04 | 1.66 | 1.60 | 0.73^e^ |
| M3-92 | 1.02 | 1.56 | 1.54 | 0.7^e^ |
| M3-93 | 0.98 | 1.71 | 1.75 | 0.8^d^ |
| M3-94 | 0.96 | 1.59 | 1.65 | 0.75^d^ |
| M3-95 | 0.95 | 1.51 | 1.59 | 0.73^e^ |
| M2-2 | 0.86 | 1.88 | 2.19 | 1^c^ |
| M3-96 | 0.83 | 1.57 | 1.89 | 0.94^c^ |
| M3-97 | 0.87 | 1.56 | 1.80 | 0.9^c^ |
| M3-98 | 0.80 | 1.58 | 1.98 | 0.98^c^ |
| M3-99 | 0.72 | 1.55 | 2.16 | 1.08^b^ |
| M3-100 | 0.73 | 1.54 | 2.10 | 1.04^c^ |
| M2-2 | 0.75 | 1.50 | 2.01 | 1^c^ |

“^a-e^” denote the significant difference (P < 0.05). There is no significant difference between samples marked with the same letter, and there is significant difference between samples marked with different letters.
